# Combined calcium, boron, and molybdenum fertilization enhances peanut biomass, yield, and economic returns in the Huaibei plain

**DOI:** 10.3389/fpls.2026.1930443

**Published:** 2026-08-05

**Authors:** Xiaohua Song, Pingli Liu, Hangyu Li, Peipei Li, Minghui Yu

**Affiliations:** 1 Xinyang Agriculture and Forestry University, Xinyang, China; 2 Hebi Agricultural and Rural Development Service Center, Hebi, China; 3 Xinyang Academy of Agricultural Sciences, Xinyang, China

**Keywords:** boron, calcium, economic benefits, micronutrient fertilizers, molybdenum, peanut, yield

## Abstract

To clarify the effects of sole and combined applications of calcium(Ca), boron(B), and molybdenum(Mo) fertilizers on peanut biomass accumulation, yield formation, and economic benefits, a field experiment was conducted in 2024 in Zhengyang County, Zhumadian City, Henan Province, China. Eight treatments were established: CK, Ca, B, Mo, Ca+B, Ca+Mo, B+Mo, and Ca+B+Mo, with three replicates for each treatment. Peanut biomass, yield components, and economic benefit indicators were determined, and the results were comprehensively evaluated using one-way analysis of variance, Tukey’s honestly significant difference(HSD) test, three-way analysis of variance, Pearson correlation analysis, and principal component analysis. The results showed that all micronutrient treatments increased peanut biomass, yield, and economic benefits to varying degrees, among which the Ca+B+Mo treatment performed the best. Under this treatment, total biomass, pod yield, kernel yield, and profit reached 11,544.60 kg ha^-1^, 5,518.35 kg ha^-1^, 3,962.10 kg ha^-1^, and 38,814.60 CNY ha^-1^, respectively, representing increases of 14.56%, 25.89%, 31.95%, and 39.72% compared with CK. One-way ANOVA indicated that all biomass, yield, and economic benefit indices differed significantly or highly significantly among treatments. Three-way ANOVA showed that Ca, B, and Mo had significant main effects on kernel yield, profit, and the benefit-cost ratio, among which Mo made the greatest contribution to total biomass, kernel yield, profit, and the benefit-cost ratio. Moreover, the B×Mo interaction had significant or highly significant effects on total biomass, kernel yield, profit, and the benefit-cost ratio. Correlation analysis showed that total biomass, kernel yield, and harvest index were all highly significantly and positively correlated with profit. Principal component analysis indicated that the first two principal components cumulatively explained 99.30% of the total variation, and the comprehensive ranking of treatments was as follows: Ca+B+Mo > Ca+Mo > Ca+B > B+Mo > Mo > B > Ca > CK. In conclusion, the combined application of Ca, B, and Mo significantly promoted peanut dry matter accumulation and improved yield and economic benefits, with the ternary combined treatment showing the best overall performance. Under the current experimental conditions, this treatment can be recommended as an optimal micronutrient fertilization strategy for peanut production in the study area; however, multi-year and multi-site validation across different cultivars, soil types, and climates is required before broader recommendations can be made.

## 1 Introduction

Peanut (Arachis hypogaea L.), as an important oilseed and cash crop in China, plays an irreplaceable strategic role in ensuring national food and edible oil security and increasing farmers’ income. According to statistics, in 2022, the peanut planting area in China was approximately 4.60 million ha, with a total production of about 18.0 million tons, accounting for 36% of global peanut production and ranking first in the world (Gelaye and Luo, 2024). The average peanut yield in China has remained stable at about 3,900 kg ha^-1^, which is markedly higher than that of India, the world’s second-largest producer, at about 1,500 kg ha^-1^, highlighting China’s advantages in intensive cultivation and improved variety breeding. In terms of regional distribution, peanut production in China is highly concentrated in the Huang-Huai-Hai Plain and southern producing areas. Among them, Henan Province has maintained a planting area of more than 1.13 million ha for many years, and Zhengyang County is widely known as the “Peanut Capital of China” (Li, 2023). However, under the current high-yield cultivation system, China’s peanut industry is facing a critical bottleneck in the transition from the traditional “high-input” model to a green and sustainable production system. For a long time, agricultural production has generally shown a fertilization pattern characterized by “emphasizing macronutrients while neglecting meso- and micronutrients” (Wang et al., 2023). Although traditional high-input intensive agriculture has ensured total output, it has also caused serious environmental problems such as nitrogen loss (Chen et al., 2021). More importantly, excessive application of nitrogen, phosphorus, and potassium (NPK) stimulates vigorous vegetative growth in the early stage of peanut development, intensifies competition for and dilution of meso- and micronutrients within plants, and consequently results in frequent problems such as luxuriant early growth but low pod-setting rate in the later stage, increased empty and unfilled pods, and reduced proportion of well-filled pods. Therefore, coordinated and balanced fertilization combining NPK with meso- and micronutrients, particularly calcium (Ca), boron (B), and molybdenum (Mo), has become a fundamental approach for overcoming the dilemma of “high yield but low efficiency” in peanut production.

A large body of empirical evidence has shown that Ca, B, and Mo play indispensable roles in peanut reproductive development, pod filling, and nitrogen metabolism. Calcium is a key element for peanut pod development, directly participating in cell wall and membrane construction as well as signal transduction. Because Ca has very poor mobility in the phloem, the Ca required after peg penetration into the soil depends almost entirely on soil supply. Studies have shown that deficiency of free Ca2+ in soil can directly induce early embryonic cell apoptosis, resulting in empty pods (Yang et al., 2017, 2020). Research on the cultivar Xianghua 2008 showed that pod yield decreased by 27.4% under high nitrogen input without Ca application (Wang et al., 2023). Zou et al. (2023) demonstrated that appropriate Ca application, such as 150 kg ha^-1^ CaO, could significantly increase the proportion of carbon assimilates allocated to pods and improve the economic coefficient (Zou et al., 2023). In addition, field experiments conducted by Xie et al. (2021), in eastern Hebei, quantified the yield-increasing effect of Ca fertilizer, showing that Ca application increased yield by 10.8%-15.3%. Zhang et al. (2020) also confirmed that a moderate Ca rate of 75 kg ha^-1^ produced the highest yield, which was significantly 9.4% and 4.7% higher than that of the no-Ca treatment in one year, and significantly 5.3% and 6.7% higher in 2018, indicating that Ca fertilizer can significantly increase the number of filled pods and 100-kernel weight (Zhang et al., 2020). Boron is likewise essential for flowering and pod setting in peanut. Meng (2024), in a field experiment, showed that in slightly B-deficient soils, basal application of 45 kg borax ha^-1^ significantly increased plant height and the number of filled pods. Li (2023) found that foliar spraying of 0.2% boric acid solution at the flowering stage effectively alleviated pollination and fertilization disorders and significantly improved pod formation rate. From a physiological perspective, Wang et al. (2025) further confirmed that B fertilizer promoted the transport and redistribution of both B and nitrogen in peanut plants, and is thus crucial for preventing hollow kernels (Zhang and Guo, 2021). Molybdenum, as an essential component of nitrate reductase and nitrogenase, directly determines the efficiency of symbiotic nitrogen fixation by rhizobia. In a fertilizer-efficiency trial conducted in Queshan County, Li and Hu (2019) reported that foliar spraying of 3.373 g ammonium molybdate per 30 m² resulted in a yield of 4,668.0 kg ha^-1^, representing an increase of 70.0 kg over the control yield of 241.2 kg ha^-1^, or 29.0%, and the yield increase was highly significant. Previous studies have also confirmed that Mo seed dressing or foliar spraying at the seedling stage can significantly increase nodule number and fresh weight and enhance nitrogenase activity by 25%-40% (Abreu-Junior et al., 2023; Gericó et al., 2020). This not only reduces dependence on external nitrogen fertilizer inputs, but also promotes the accumulation of protein, chlorophyll, and soluble sugars in plants, thereby providing a material basis for high yield.

Although the effects of individual application of these meso- and micronutrients have been widely demonstrated, peanut, as a crop highly sensitive to reproductive organ development, depends more strongly on synergistic interactions among multiple nutrients to achieve high yield and quality. Jiang et al. (2017) clearly pointed out that the synergistic effects of elements such as Ca, Mo, and B exert an integrated influence on the coordination of vegetative and reproductive growth in peanut. Relevant studies have shown that scientifically combining Ca, B, and Mo can generate substantial synergistic effects far exceeding those of single-element application. Several field experiments have confirmed that a reasonable combined application scheme of Ca, B, and Mo, for example, basal application of 150 kg ha^-1^ Ca fertilizer together with foliar spraying of 0.5 kg ha^-1^ B and 0.8 kg ha^-1^ Mo, can increase peanut yield by 16.3%-23.7% compared with the control (Jiang et al., 2017). Liang (2018) also suggested the significant benefits of combined application of organic Ca, Mg, Zn, B, and Mo. Mechanistically, Ca improves the root-zone environment, B optimizes assimilate partitioning, and Mo strengthens nitrogen metabolism; together, these three elements form an efficient “carbon-nitrogen-structure” synergistic network (Garrone et al., 2025; Xu et al., 2026). However, under the sandy loam conditions of the Huang-Huai-Hai Plain, especially in major peanut-producing areas of Henan Province, systematic empirical research and quantitative analysis on the main effects and interaction effects of combined Ca, B, and Mo application, particularly comprehensive evaluation based on multiple indicators, remain limited. Thus, we hypothesized that (i) combined application of Ca, B, and Mo would outperform sole applications for peanut biomass accumulation, yield formation, and economic returns; and (ii) Mo would be the dominant factor among the three nutrients, given that the available Mo at the experimental site (0.12 mg kg^-1^) was below the critical level for peanut, whereas available Ca and B were relatively adequate.

To address these knowledge gaps, a field experiment was conducted in 2024 in Zhengyang County, Henan Province, using the peanut cultivar Yuhua 9326. Eight treatments were established to evaluate the effects of sole and combined meso- and micronutrient application on peanut biomass, yield, and economic benefits. The findings aim to provide a scientific basis for optimizing meso- and micronutrient fertilization strategies for peanut production in the Huaibei Plain.

## 2 Materials and methods

### 2.1 Experimental site

The field experiment was conducted in 2024 in Xiongzhai Town, Zhengyang County, Zhumadian City, Henan Province, China. The experimental site is located in the Huaibei Plain, with flat terrain and an elevation of approximately 65 m. It belongs to a monsoon climate zone in the transitional region from warm temperate to subtropical climates, with a mean annual temperature of 14.9°C, annual precipitation of 960 mm, and a frost-free period of 228 d. The previous crop was wheat, and the field followed a wheat-peanut double-cropping rotation system with good irrigation and drainage conditions and a well-developed drainage ditch network. The tested soil was sandy loam. Before the experiment, soil samples were collected from the 0–20 cm plow layer, air-dried, ground, and sieved to determine basic physicochemical properties. The results were as follows: pH 6.2, organic matter 12.5 g kg^-1^, total nitrogen 0.85 g kg^-1^, alkali-hydrolyzable nitrogen 68 mg kg^-1^, available phosphorus 32 mg kg^-1^, available potassium 98 mg kg^-1^, available calcium 850 mg kg^-1^, available boron 0.35 mg kg^-1^, and available molybdenum 0.12 mg kg^-1^.

### 2.2 Experimental materials

The tested peanut cultivar was Yuhua 9326, an erect and sparsely branched large-seeded peanut cultivar with a growth period of approximately 120 d, suitable for both spring sowing and summer sowing after wheat harvest in Henan Province. The fertilizers used in the experiment were as follows: calcium fertilizer was calcium nitrate [Ca(NO_3_)_2_·4H_2_O, containing 19% Ca], applied as a basal fertilizer; boron fertilizer was borax [Na_2_B_4_O_7_·10H_2_O, containing 11% B], applied as a seed treatment; and molybdenum fertilizer was ammonium molybdate [(NH_4_)_6_Mo_7_O_2__4_·4H_2_O, containing 54% Mo], also applied as a seed treatment. Basal fertilization followed the local high-yield cultivation practice: a compound fertilizer (N-P2O5-K2O, 15-15-15) was applied at 525 kg ha^-1^ before land preparation, and urea was topdressed at the pegging stage at 75 kg ha^-1^. Field management practices included fine rotary tillage before sowing to achieve a loose upper layer, firm lower layer, and level soil surface; plastic film mulching cultivation; conventional control of diseases, insects, and weeds during the growing season; timely irrigation during drought; and prompt drainage after rainfall. Except for the experimental factors, all other management conditions were kept strictly consistent across treatments.

### 2.3 Experimental design

The experiment was designed by combining single-factor treatments with a factorial structure. A total of eight treatments were established: CK, Ca, B, Mo, Ca+B, Ca+Mo, B+Mo, and Ca+B+Mo, with three replicates per treatment, resulting in 24 experimental plots in total. For statistical analysis, the treatments were further coded into three binary factors, namely Ca, B, and Mo (0 = not applied, 1 = applied), in order to test the main effects and interaction effects of each factor. The detailed fertilization schemes were as follows: CK, no meso- or micronutrient fertilizer application, with only basal fertilization; Ca, sole application of calcium fertilizer, with calcium nitrate applied basally at 395 kg ha^-1^ (equivalent to 75 kg Ca ha^-1^); B, sole application of boron fertilizer, with borax used as a seed treatment at 4.5 kg ha^-1^ (equivalent to 0.5 kg B ha^-1^); Mo, sole application of molybdenum fertilizer, with ammonium molybdate used as a seed treatment at 0.56 kg ha^-1^ (equivalent to 0.3 kg Mo ha^-1^); and Ca+B, Ca+Mo, B+Mo, and Ca+B+Mo, corresponding combined treatments applied at the same rates as above. Each plot measured 24 m² (6 m×4 m) and was arranged in a randomized block design, with a 1 m walkway between blocks and 0.5 m ridges between plots for isolation. The planting density was 165,000 planting holes ha^-1^, with row spacing of 40 cm and hole spacing of 15 cm, and two seeds were sown per hole. Sowing was carried out on April 25, 2024, and harvesting took place on September 5, 2024.

### 2.4 Determination of indices and methods

At maturity, peanut plants were sampled by plot. Fifteen representative plants were selected from each plot, and the fresh weights of roots, stems and leaves, shells, and kernels were measured separately. Samples were first heated at 105°C for 30 min to deactivate enzymes and then oven-dried at 80°C to constant weight. Dry matter weight was calculated, and the aboveground biomass, belowground biomass, and total biomass were then obtained by summation. Pod yield, kernel yield, shelling percentage, and harvest index were also determined. Shelling percentage was calculated as the percentage of kernel yield relative to pod yield, and harvest index was calculated as the ratio of economic yield to total biomass.

Economic benefit analysis included output value, cost, profit, and benefit-cost ratio. Production costs included expenditures on fertilizers, seeds, mechanized operations, labor management, pesticides, and other agricultural inputs. Profit was calculated as output value minus production cost, and the benefit-cost ratio was calculated as the ratio of output value to cost.

### 2.5 Data processing and statistical analysis

Biomass, yield, and economic benefit data were combined, and each individual plot replicate was treated as a statistical unit. Descriptive statistics were first performed for all variables, and the results were expressed as mean ± SD. Subsequently, one-way analysis of variance (one-way ANOVA) was conducted with treatment as the fixed factor, followed by Tukey’s honestly significant difference (HSD) test for multiple comparisons. Different lowercase letters indicate significant differences among treatments at *P* < 0.05. On this basis, treatments were further coded into three binary factors, Ca, B, and Mo, and a three-way ANOVA model was constructed as follows: trait ~ Ca × B × Mo (which in R formula notation expands to all main effects and all two- and three-way interactions: Ca + B + Mo + Ca:B + Ca: Mo + B:Mo + Ca:B:Mo), to test the main effects and interaction effects. Pearson correlation analysis was then performed using total biomass, pod yield, kernel yield, shelling percentage, harvest index, profit, and benefit-cost ratio. In addition, based on the mean values of each treatment, principal component analysis (PCA) was conducted on total biomass, kernel yield, shelling percentage, harvest index, profit, and benefit-cost ratio. The first two principal components were weighted by their contribution rates to calculate comprehensive scores, which were then used to comprehensively evaluate the different fertilization combinations. All statistical analyses were conducted in the R software environment.

## 3 Results and analysis

### 3.1 Effects of different treatments on peanut biomass

As shown in **Table 1**, all biomass indices of peanut were improved to varying degrees under different treatments. Total biomass was highest under the Ca+B+Mo treatment, reaching 11,544.60 kg ha^-1^, which was 14.56% higher than that under CK (10,077.15 kg ha^-1^). This was followed by the Ca+Mo and Mo treatments, which reached 11,544.60 and 11,096.85 kg ha^-1^, respectively. Sole application of Ca, B, or Mo promoted the accumulation of root, stem and leaf, and aboveground biomass, while the promotive effects were further enhanced under combined application treatments, indicating that combined application of meso- and micronutrients was more conducive to dry matter formation and accumulation in peanut plants.

**Table 1:** Mean values, standard deviations, and significance groupings of peanut biomass indices under different treatments.

Treatment	Root biomass (kg ha^-1^)	Stem and leaf biomass (kg ha^-1^)	Aboveground biomass (kg ha^-1^)	Total biomass (kg ha^-1^)
CK	604.65 ± 9.90 d	4,030.80 ± 66.30 d	9,472.35 ± 155.85 d	10,077.15 ± 165.75 d
Ca	641.70 ± 13.20 c	4,278.00 ± 87.90 c	10,053.30 ± 206.85 c	10,695.00 ± 220.05 c
B	637.95 ± 6.90 c	4,252.80 ± 45.45 c	9,994.05 ± 106.80 c	10,631.85 ± 113.40 c
Mo	665.85 ± 9.00 abc	4,438.80 ± 60.30 abc	10,431.00 ± 141.60 abc	11,096.85 ± 150.60 abc
Ca+B	663.90 ± 8.40 abc	4,425.60 ± 55.65 abc	10,400.10 ± 130.80 abc	11,064.00 ± 139.20 abc
Ca+Mo	686.40 ± 4.65 ab	4,575.75 ± 31.20 ab	10,753.20 ± 73.05 ab	11,439.60 ± 77.70 ab
B+Mo	662.10 ± 8.10 bc	4,413.30 ± 53.55 bc	10,371.45 ± 125.85 bc	11,033.40 ± 133.95 bc
Ca+B+Mo	692.70 ± 18.30 a	4,617.90 ± 121.80 a	10,851.90 ± 286.35 a	11,544.60 ± 304.50 a
F-value	21.69	21.65	21.65	21.65
P-value	<0.001	<0.001	<0.001	<0.001

Different lowercase letters within the same column indicate significant differences among treatments at *P* < 0.05. The F- and P-values are the results of one-way ANOVA.

One-way ANOVA showed that root biomass, stem and leaf biomass, aboveground biomass, and total biomass all differed extremely significantly among treatments (*P* < 0.01) (**Table 1**). Three-way ANOVA further indicated that total biomass was mainly affected by Ca (*P* < 0.001), B (*P* = 0.004), Mo (*P* < 0.001), and the B×Mo interaction (*P* = 0.007) (**Supplementary Table 1**). Among these factors, Mo showed the highest contribution rate, indicating that molybdenum fertilizer exerted the strongest driving effect on biomass accumulation.

### 3.2 Effects of different treatments on peanut yield formation

As shown in **Table 2**, peanut yield and its component traits differed markedly among treatments. The Ca+B+Mo treatment produced the highest pod yield and kernel yield, reaching 5,518.35 kg ha^-1^ and 3,962.10 kg ha^-1^, respectively, which were 25.89% and 31.95% higher than those under CK. Meanwhile, the Ca+B+Mo treatment achieved a shelling percentage of 71.97% and a harvest index of 0.474, indicating that the combined application of meso- and micronutrient fertilizers not only increased yield level, but also promoted kernel filling and the allocation of dry matter to economic organs.

**Table 2:** Mean values, standard deviations, and significance groupings of peanut yield and yield component indices under different treatments.

Treatment	Pod yield (kg ha^-1^)	Kernel yield (kg ha^-1^)	Shelling percentage(%)	Harvest index
CK	4,383.60 ± 72.15 d	3,002.70 ± 49.35 d	68.72 ± 0.60 d	0.43 ± 0.01 d
Ca	4,791.30 ± 98.55 c	3,315.60 ± 68.25 c	68.76 ± 0.20 d	0.45 ± 0.01 bcd
B	4,784.40 ± 51.00 c	3,325.20 ± 35.55 c	69.44 ± 0.40 cd	0.45 ± 0.00 cd
Mo	5,082.30 ± 69.00 b	3,567.75 ± 48.45 b	70.31 ± 0.56 bc	0.45 ± 0.01 bc
Ca+B	5,144.70 ± 64.65 b	3,642.45 ± 45.90 b	70.62 ± 0.66 abc	0.46 ± 0.00 ab
Ca+Mo	5,376.60 ± 36.60 a	3,828.15 ± 25.95 a	71.17 ± 0.58 ab	0.48 ± 0.00 a
B+Mo	5,097.45 ± 61.95 b	3,593.70 ± 43.65 b	70.38 ± 0.64 bc	0.46 ± 0.01 abc
Ca+B+Mo	5,518.35 ± 145.65 a	3,962.10 ± 104.55 a	71.97 ± 0.42 a	0.47 ± 0.00 a
F-value	59.31	86.01	14.20	17.19
P-value	<0.001	<0.001	<0.001	<0.001

Different lowercase letters within the same column indicate significant differences among treatments at *P* < 0.05. The F- and P-values are the results of one-way ANOVA.

The results of one-way ANOVA showed that pod yield, kernel yield, shelling percentage, and harvest index all differed extremely significantly among treatments (*P* < 0.01). Three-way ANOVA further showed that kernel yield was affected by Ca (*P* < 0.001), B (*P* < 0.001), Mo (*P* < 0.001), and the B×Mo interaction (*P* < 0.001). Shelling percentage was affected by Ca (*P* < 0.001), B (*P* < 0.001), Mo (*P* < 0.001), and the Ca×B interaction (*P* = 0.044) (**Supplementary Table 1**). Harvest index was affected by Ca (*P* < 0.001), B (*P* = 0.003), Mo (*P* < 0.001), and the B×Mo interaction (*P* = 0.015). Overall, Mo remained the main-effect factor contributing most strongly to yield formation.

### 3.3 Effects of different treatments on the economic benefits of peanut

As shown in **Table 3**, the output value, profit, and benefit-cost ratio of peanut exhibited consistent trends across treatments. The Ca+B+Mo treatment achieved the best economic performance, with a profit of 38,814.60 CNY ha^-1^, which was 39.72% higher than that of CK (27,781.20 CNY ha^-1^). The benefit-cost ratio also increased from 5.12 under CK to 6.75 under Ca+B+Mo. The pairwise combined application treatments also showed relatively high economic returns, among which the Ca+Mo treatment ranked second, indicating that the combined application of meso- and micronutrients could significantly improve economic returns while increasing yield.

**Table 3:** Mean values, standard deviations, and significance groupings of economic benefit indices of peanut under different treatments.

Treatment	Output value (CNY ha^-1^)	Profit (CNY ha^-1^)	Benefit-cost ratio
CK	34,531.20 ± 568.05 d	27,781.20 ± 568.05 d	5.12 ± 0.08 d
Ca	38,129.70 ± 784.65 c	31,379.70 ± 784.65 c	5.65 ± 0.12 c
B	38,239.05 ± 408.15 c	31,489.05 ± 408.15 c	5.67 ± 0.06 c
Mo	41,029.80 ± 556.95 b	34,279.80 ± 556.95 b	6.08 ± 0.08 b
Ca+B	41,888.55 ± 526.80 b	35,138.55 ± 526.80 b	6.20 ± 0.08 b
Ca+Mo	44,023.50 ± 298.80 a	37,273.50 ± 298.80 a	6.52 ± 0.05 a
B+Mo	41,327.40 ± 501.60 b	34,577.40 ± 501.60 b	6.12 ± 0.08 b
Ca+B+Mo	45,564.60 ± 1,202.10 a	38,814.60 ± 1,202.10 a	6.75 ± 0.18 a
F-value	86.00	86.00	87.90
P-value	<0.001	<0.001	<0.001

Different lowercase letters within the same column indicate significant differences among treatments at *P* < 0.05. The F-value and P-value were obtained from one-way ANOVA.

One-way ANOVA showed that output value, profit, and benefit-cost ratio all differed extremely significantly among treatments (*P* < 0.01). Three-way ANOVA further indicated that profit was affected by Ca (*P* < 0.001), B (*P* < 0.001), Mo (*P* < 0.001), and the B×Mo interaction (*P* < 0.001). The benefit-cost ratio was also affected by Ca (*P* < 0.001), B (*P* < 0.001), Mo (*P* < 0.001), and the B×Mo interaction (*P* < 0.001) (**Supplementary Table 1**). The overall effects were pronounced, and the Mo factor showed the highest contribution rate, indicating that molybdenum fertilizer played a key role in increasing both yield and economic benefits.

### 3.4 Correlation analysis

**Figure 1:**
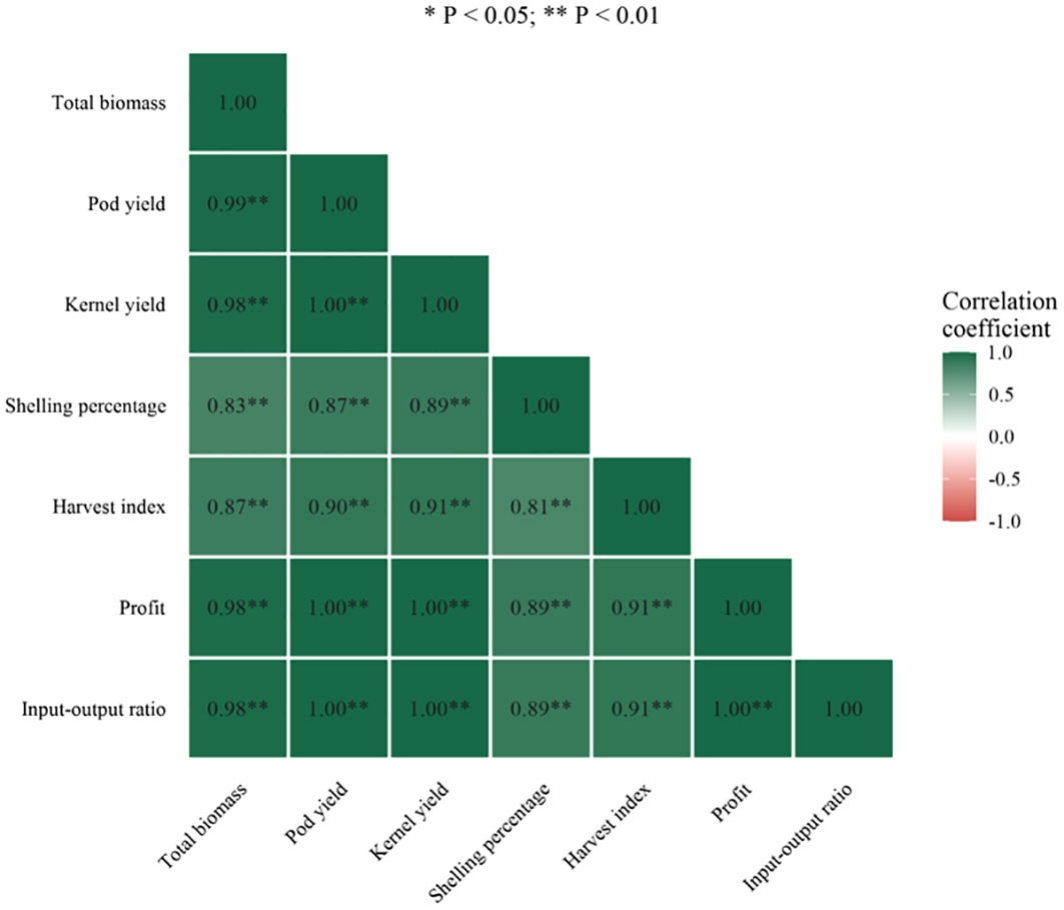
Correlation heatmap of peanut biomass, yield, and economic benefit indices. Darker colors in the figure indicate stronger correlations, and green indicates positive correlations. ^##^*P<0.05; **P<0.01.

The correlation analysis showed that total biomass (kg ha^-1^) was highly significantly and positively correlated with kernel yield (kg ha^-1^) (r = 0.980, *P* < 0.001), and also highly significantly and positively correlated with profit (CNY ha^-1^) (r = 0.980, *P* < 0.001). Kernel yield (kg ha^-1^) was highly significantly and positively correlated with profit (CNY ha^-1^) (r =1.000, *P* < 0.001). In addition, shelling percentage (%) was highly significantly and positively correlated with profit (CNY ha^-1^) (r = 0.886, *P* < 0.001), and harvest index was also highly significantly and positively correlated with profit (CNY ha^-1^) (r = 0.909, *P* < 0.001). These results indicate a strong consistency among biomass accumulation, kernel formation, and economic returns, with kernel yield in particular playing a direct role in increasing profit (**Figure 1**).

### 3.5 Comprehensive evaluation by principal component analysis

**Figure 2:**
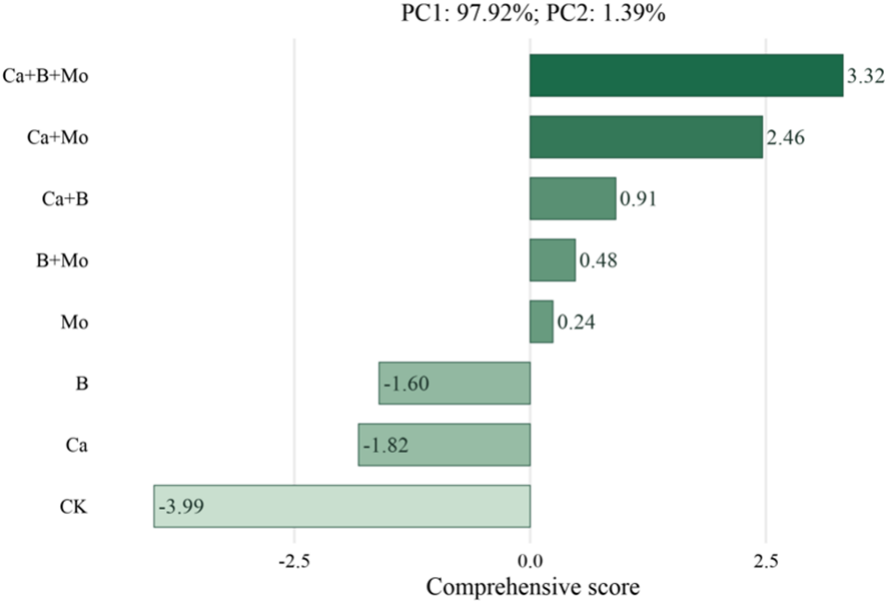
Ranking of principal component analysis scores for the overall performance of peanut under different treatments. Note: The comprehensive score was calculated by weighting the first two principal components according to their contribution rates. A higher score indicates better overall performance of the treatment.

Based on the single-index analysis, principal component analysis (PCA) was further used to comprehensively evaluate peanut biomass, yield, and economic benefit indices under different treatments. The results showed that the first two principal components cumulatively explained 99.30% of the total variation, indicating that they could effectively represent the overall performance of the treatments. The ranking of comprehensive scores is shown in **Figure 2**. Among all treatments, Ca+B+Mo had the highest comprehensive score, indicating that it was the optimal fertilization combination under the experimental conditions. The Ca+Mo and Ca+B treatments ranked second and third, respectively, and also exhibited relatively good overall performance.

## 4 Discussion

### 4.1 Effects of combined meso- and micronutrient fertilization on peanut biomass accumulation and possible mechanistic interpretations

The results of this study showed that different meso- and micronutrient fertilizer treatments increased root, stem and leaf, aboveground, and total biomass of peanut to varying degrees, among which the Ca+B+Mo treatment produced the greatest increase. This result is consistent with that reported by Jiang et al. (2017), indicating that the combined application of meso- and micronutrients exerts a synergistic effect on peanut dry matter accumulation. From the perspective of plant nutritional physiology, calcium helps maintain the structural stability of cell walls and cell membranes and promotes normal root elongation and absorptive function (Palta, 2010). Boron, in contrast, forms borate diester bonds with cell wall polysaccharides, especially rhamnogalacturonan II, thereby ensuring the structural integrity of cell walls in newly formed tissues and promoting efficient sucrose transport in the phloem, which directly affects floral development and seed filling (Gupta and Macleod, 2018). Although molybdenum is required only in trace amounts, it is the core component of nitrate reductase and the iron-molybdenum cofactor (FeMo-co) of nitrogenase, and thus plays an irreplaceable catalytic role in nitrate assimilation and symbiotic nitrogen fixation (Oliveira et al., 2022). When these three nutrients were applied in combination, they not only improved root vigor and nutrient uptake, but also promoted assimilate synthesis and translocation, thereby resulting in a strong advantage in biomass accumulation.

The three-way ANOVA in this study showed that Mo had the highest contribution rate to total biomass, and that the B×Mo interaction was extremely significant, indicating that under the present experimental conditions, molybdenum was not only an important limiting factor for biomass formation, but that its beneficial effect may also depend on coordinated support from boron nutrition. Mechanistically, based on literature evidence, molybdenum, as a key component of nitrate reductase (NR) and nitrogenase, regulates nitrogen assimilation efficiency and the nitrogen-fixing capacity of legumes. Under molybdenum deficiency, nitrate reduction may be restricted even when soil nitrogen is sufficient, thereby hindering nitrogen metabolism and ultimately limiting protein synthesis and plant growth (Banerjee and Nath, 2021; Kovács et al., 2015). However, whether the “nitrogen assimilation output” driven by molybdenum can be translated into increased biomass also depends on the availability of carbon skeletons and the capacity for assimilate partitioning. Boron supports this process by maintaining cell wall structure and ensuring the transport of photosynthates such as sucrose in the phloem. Long and Peng (2023) found that boron deficiency restricted photosynthate transport and disrupted source-sink balance, thereby weakening the realization of molybdenum effects. Therefore, under combined application, the coupling of B and Mo often leads to a stronger growth response. Previous studies have reported that the B+Mo treatment increased yield by 12.3%-18.7% compared with single application, while aboveground biomass increased by 36.5%, significantly higher than the 19.2% increase under sole Mo application. This was accompanied by synchronous increases in leaf nitrate reductase activity and sucrose transport rate, supporting the synergistic mechanism in which “Mo drives nitrogen metabolism, whereas B ensures carbon transport and allocation” (Gericó et al., 2020; Steiner et al., 2023; Zheng et al., 2013). Therefore, the highest contribution rate of Mo and the significant B×Mo interaction are not contradictory: the former reflects the dominant limiting role of Mo, whereas the latter emphasizes that relatively coordinated boron nutrition is necessary to maximize the beneficial effects of molybdenum. This finding suggests that efficient peanut fertilization should shift from single-nutrient supplementation toward integrated application strategies based on nutrient interactions.

### 4.2 Effects of meso- and micronutrient fertilizers on peanut yield formation and kernel filling

Peanut yield formation depends not only on early vegetative growth, but also closely on pod development, kernel filling, and the efficiency of dry matter allocation to economic organs. In this study, the Ca+B+Mo treatment achieved the highest pod yield, kernel yield, shelling percentage, and harvest index, and kernel yield was 31.95% higher than that of CK. These results indicate that the core role of combined meso- and micronutrient application in increasing peanut yield lies not merely in promoting vegetative growth, but more importantly in enhancing pod maturation and kernel filling, thereby improving yield components and product formation efficiency. Based on published literature, Ca mainly provides the structural and signaling basis for pod and seed development. Its uptake depends largely on direct soil supply rather than phloem transport, and it participates in cell wall formation, membrane stability, signal transduction, and embryo development. Calcium deficiency can lead to embryo abortion and abnormal pod development, thereby reducing yield (El-Temsah et al., 2023). Accordingly, Ca application can significantly promote the growth of peanut cultivar Nonghua 5 and increase pod number per plant, shelling percentage, and 100-kernel weight (Jiang et al., 2017). In acidic red soil, application of 568 kg CaO ha^-1^ resulted in the highest kernel yield of 3,664 kg ha^-1^, representing an 18.7% increase over the control (Wang et al., 2023). In addition, nano-calcium and conventional calcium fertilizer increased pod yield by approximately 37.3% and 29.4%, respectively (El-Temsah et al., 2023), while yield increases among different cultivars ranged from 3.3% to 10.9% (Hamza et al., 2023), with simultaneous improvements in shelling performance and stress resistance. In contrast, B mainly regulates pollen germination and pollen tube elongation, thereby ensuring pollination, fertilization, and kernel filling. In nano-/biofertilizer systems, foliar spraying of boron at 200 ppm increased kernel yield by 7.3%, and this increase reached 10.8% when combined with calcium (Abdelghany et al., 2022). Mo, by participating in nitrate reductase and nitrogenase systems, regulates nitrogen metabolism and promotes rhizobial symbiotic nitrogen fixation, thereby improving nitrogen nutrition and facilitating kernel formation. For example, seed treatment with Co+Mo significantly increased peanut yield (Gericó et al., 2020), while foliar spraying of Mo increased pod yield by about 10% and 8% in two growing seasons of IAC Tatu ST, respectively (Steiner et al., 2023). Overall, Ca mainly provides the “structural foundation” for pod development, B safeguards the “reproductive process,” and Mo strengthens the “nitrogen metabolic supply,” and together they shape pod formation, kernel filling, and dry matter partitioning.

The statistical results further support the above mechanistic interpretation and indicate that meso- and micronutrients do not act independently. Three-way ANOVA showed that Ca, B, and Mo all had extremely significant main effects on kernel yield, while the B×Mo interaction was also extremely significant. In addition, Ca×B had a significant effect on shelling percentage. Jiang et al. (2017) found in a field experiment with Nonghua 5 that the combined application of Ca, Mo, and B increased peanut kernel yield by 16.3% compared with the control without meso- and micronutrient application, and this increase was greater than that under sole Ca application, which was approximately 10%. In their study, treatment T2 (Ca+Mo) already showed significantly higher kernel yield than the control, reaching 3,587.2 kg ha^-1^, whereas the addition of B in T4 (Ca+Mo+B) further increased kernel yield to 3,720.5 kg ha^-1^ (Jiang et al., 2017). These results suggest that the nitrogen metabolism and nitrogen fixation capacity strengthened by Mo and the pollen viability and fertilization processes supported by B may generate a “coupled gain” during the reproductive stage, thereby enhancing kernel filling and final kernel yield (Gericó et al., 2020; Steiner et al., 2023). Meanwhile, the significant effect of Ca×B on shelling percentage also supports a synergistic logic extending “from development to partitioning.” In experiments conducted in 2013 and 2014, the shelling percentage under Ca+Mo+B reached 74.3% and 75.1%, respectively, significantly higher than the 69.5% and 69.9% of the control (Jiang et al., 2017). Given that Ca is responsible for pod structural integrity and kernel development, whereas B is involved in pollen viability and successful fertilization, it can be inferred that balanced supply of these nutrients helps ensure the smooth progression of the entire process from flowering to pod setting and subsequent kernel filling (Galeriani et al., 2022). Overall, these results are consistent with the theory of “synergy-antagonism/nutrient balance.” For peanut, a crop highly sensitive to reproductive organ development, balanced combined application of Ca, B, and Mo, rather than single-nutrient supplementation, is more likely to simultaneously optimize pod formation, kernel filling, and dry matter allocation, thereby more fully releasing yield potential and improving quality.

### 4.3 Consistency between yield increase and efficiency improvement of meso- and micronutrient fertilization and its practical implications

In agricultural production, whether a yield-increasing measure can simultaneously improve economic returns is a key criterion for evaluating the feasibility of fertilization technologies for wider adoption. In this study, the trends in output value, profit, and benefit-cost ratio were basically consistent with the yield results, and profit was highly significantly and positively correlated with kernel yield. This indicates that the increase in returns under meso- and micronutrient treatments was mainly derived from improved economic yield rather than simply from price fluctuations or cost reduction. Compared with CK, the Ca+B+Mo treatment increased profit by 39.72% and also achieved the highest benefit-cost ratio, indicating that this ternary combination not only had a clear yield-enhancing effect under the current experimental conditions, but also demonstrated high economic feasibility.

These findings have important implications for practical production. At present, some farmers still focus mainly on macronutrient inputs while neglecting supplementation of meso- and micronutrients, which often leads to the problem that “N, P, and K are sufficient, but yield and quality remain difficult to improve.” This study suggests that, under relatively stable basal nutrient supply, appropriate supplementation of Ca, B, and Mo, especially in combined application, is more conducive to simultaneously increasing biomass, yield, and economic returns. Therefore, in high-yield and high-efficiency peanut cultivation systems, meso- and micronutrient fertilizers should not be regarded as optional auxiliary inputs, but rather as an important component of optimized nutrient management.

### 4.4 Interpretation of factor effects and comprehensive evaluation results

Unlike simple comparisons of treatment means, three-way ANOVA further revealed the relative importance of each nutrient factor and their interactions. In this study, Mo showed a dominant effect on key indicators such as total biomass, kernel yield, profit, and benefit-cost ratio, indicating that under the present experimental conditions, molybdenum may be the key factor limiting further improvement in the overall performance of peanut. This result is consistent with the fundamental role of molybdenum in biological nitrogen fixation and nitrogen assimilation. Molybdenum is the core component of the iron-molybdenum cofactor (FeMo-cofactor) required for nitrogenase activity, ensuring that rhizobia in root nodules reduce atmospheric nitrogen to available ammonia, thereby improving nitrogen supply efficiency (Crusciol et al., 2019). At the same time, molybdenum also promotes nitrogen assimilation and the absorption, transport, and utilization of nitrogen by supporting nitrate reductase activity (Gericó et al., 2020). Previous studies have also demonstrated the effectiveness of molybdenum supplementation at the agronomic and yield levels, such as by increasing nodule formation and nitrogen fixation efficiency, as well as improving pod number and kernel yield, particularly in acidic soils or under low-Mo supply conditions (Kundu et al., 2023). Therefore, the significant contribution of Mo in this study not only reflects its regulatory role in key nitrogen metabolic processes, but also suggests that molybdenum supplementation is an important approach for improving peanut growth, yield formation efficiency, and ultimately economic returns (Banerjee et al., 2021; Kundu et al., 2023).

In addition, correlation analysis showed that total biomass was highly significantly and positively correlated with both kernel yield and profit, while shelling percentage and harvest index were also significantly and positively correlated with profit. These results indicate a strong coupling relationship among vegetative growth, kernel formation, and economic benefits. Principal component analysis further integrated multiple individual indicators into a comprehensive score. The ranking results showed that the Ca+B+Mo treatment had the best overall performance, followed by Ca+Mo and Ca+B. In other words, from individual indicators to comprehensive multi-index evaluation, the determination of the optimal treatment was highly consistent, which strengthens the robustness and interpretability of the study conclusions.

### 4.5 Study limitations and future perspectives

Although this study systematically evaluated the effects of sole and combined application of calcium, boron, and molybdenum on peanut biomass, yield, and economic benefits, several limitations remain. First, the current data were mainly derived from a single-site experiment, lacking repeated validation across different ecological regions, soil types, and years. Therefore, the regional applicability of the conclusions still requires further verification. Second, this study focused primarily on yield and economic outcomes, with relatively limited investigation of mechanistic indicators such as leaf nutrient status, nodule number, photosynthetic characteristics, enzyme activities, and kernel quality. As a result, it is still insufficient to fully explain the synergistic mechanisms of different meso- and micronutrients at a deeper level. Third, the economic benefit analysis was based on specific cost and price conditions, and market fluctuations may influence the ranking of returns to a certain extent.

Future research can be further deepened in three aspects. First, multi-site and multi-year long-term experiments should be conducted to evaluate the stability of the yield-increasing effects of combined Ca, B, and Mo application under different regional conditions and background fertility levels. Second, physiological, biochemical, and nutrient diagnostic indicators should be integrated to clarify the key mechanisms by which meso- and micronutrients affect peanut root vigor, nitrogen metabolism, pod formation, and kernel filling. Third, fertilization effects should be further linked with input-output analysis, quality evaluation, and green production goals, so as to develop more practical and widely applicable precision application models for meso- and micronutrient fertilizers. Such efforts would not only deepen the research, but also enhance the applicability of the findings in agricultural production.

## 5 Conclusions

Application of Ca, B, and Mo fertilizers increased peanut biomass, yield, and economic benefits, with combined applications outperforming sole applications and Ca+B+Mo showing the best overall performance. Three-way ANOVA identified Mo as the dominant factor and revealed significant B×Mo synergistic effects on key traits. Correlation and principal component analyses confirmed that Ca+B+Mo was the optimal combination under the present single-site, single-year conditions, providing an effective fertilization strategy for peanut on sandy loam in Zhengyang County.

Several limitations should be acknowledged. As a single-site, single-year experiment with one cultivar and three replicates, the conclusions are restricted to the current conditions. Moreover, the mechanistic interpretations remain inferential. Future work should extend the Ca+B+Mo strategy across diverse environments, couple yield responses with mechanistic and quality measurements, and refine regionally adaptable fertilization models for peanut.

## Data Availability

The raw data supporting the conclusions of this article will be made available by the authors, without undue reservation.
