# Supplementary material for: Combined calcium, boron, and molybdenum fertilization enhances peanut biomass, yield, and economic returns in the Huaibei plain
Source: Front Plant Sci. 2026 Aug 5;17:1930443. doi: 10.3389/fpls.2026.1930443 (PMC13485961; doi:10.3389/fpls.2026.1930443)
Supplement: Supplementary file 1 [file Table1.docx]

**Table S1 Summary of three-way ANOVA results (P-values and significance) for peanut biomass, yield, and economic benefit indices**

| **Trait** | **Ca (P)** | **B (P)** | **Mo (P)** | **Ca×B (P)** | **Ca×Mo (P)** | **B×Mo (P)** |
| --- | --- | --- | --- | --- | --- | --- |
| Total biomass | <0.001 *** | 0.004 ** | <0.001 *** | ns | ns | 0.007 ** |
| Kernel yield | <0.001 *** | <0.001 *** | <0.001 *** | ns | ns | <0.001 *** |
| Shelling percentage | <0.001 *** | <0.001 *** | <0.001 *** | 0.044 * | ns | ns |
| Harvest index | <0.001 *** | 0.003 ** | <0.001 *** | ns | ns | 0.015 * |
| Profit | <0.001 *** | <0.001 *** | <0.001 *** | ns | ns | <0.001 *** |
| Benefit-cost ratio | <0.001 *** | <0.001 *** | <0.001 *** | ns | ns | <0.001 *** |

**Note: Effects ns in the text are marked as “ns”. “***” denotes P < 0.001; “**” denotes P < 0.01; “*” denotes P < 0.05.**
